# GENDER DIFFERENCES IN CARE USE IN THE NETHERLANDS: CHANGES BETWEEN 1995 AND 2016

**DOI:** 10.1093/geroni/igz038.1956

**Published:** 2019-11-08

**Authors:** Mari Aaltonen, Dorly Deeg, Marjolein Broese van Groenou

**Affiliations:** 1 Tampere University, Faculty of Social Sciences, Tampere, Finland; 2 Amsterdam University Medical Centres, location VU University Medical Center, Department of Epidemiology and Biostatistics, Amsterdam, Netherlands; 3 VU University Amsterdam, Faculty of Social Sciences, Amsterdam, Netherlands

## Abstract

In recent decades, care policy in the Netherlands reduced budgets for residential care and formal home care, which increased the demand for informal care. Women use formal care more often than men, but we lack information on the extent to which the gender gap in care use is explained by differences in individual chracteristics and changes in care policy. Data from the Longitudinal Aging Study Amsterdam (LASA) were employed to explore the gender gap in the use of informal, formal and private home care, community services, and residential care in the years 1996-2016, analyzed using Generalized Estimating Equations (GEE). The data consisted of 9,497 observations, gathered from 3,369 respondents aged 65-85. Women used all types of formal care more than men. The gender differences persisted even when individual characteristics were taken into account; however, only in residential care the differences diminished after care preferences were included in the analysis. During the study years, the gender gap increased in formal home care and in non-use of care, as women increasingly used formal home care and the proportion of men without care expanded. The gender gap in informal care use reversed, with men using more informal care during the earlier years and women using more in the later years. The persistent and even increasing gender differences in care use deserve further exploration of the role of gender in current care culture. The growing gender gab in non-use of care raises concern for older men and their possible increase in unmet care needs.

